# Hospital Meetings

**Published:** 1903-05-09

**Authors:** 


### HOSPITAL MEETINGS.

### HAMPSTEAD GENERAL HOSPITAL.

An extraordinary general meeting was held at this hospital on April 30th for the purpose of considering and confirming the resolutions passed at the meeting held on April 4th, having reference to the change of name of the institute and the alterations arising therefrom to be made in the articles of association. The chair was taken by Mr. W. F. Malcolm, who put the two resolutions to the meeting, which, being carried unanimously, the proceedings came to a close. The hospital, therefore, will in future be known as the Hampstead General Hospital.

### THE BOLINGBROKE HOSPITAL (INCORPORATED).

The annual general meeting of the Governors of this hospital was held on May 1st, Canon Erskine Clarke being in the chair. The report and audited accounts for 1902 having been received and adopted, a somewhat lengthy discussion took place anent the date of the annual meeting, which it was finally resolved should be held before April 30th in future. Mr. E. G. Thorne alluded with much regret to the retirement of Mr. Cecil Lyter from the post of medical superintendent of the hospital, owing to his having joined the Middlesex Hospital; he was, however, elected upon the Board of Management. In replying to a vote of thanks, the Chairman sketched briefly the history of the hospital of which he is the founder, and showed how, with some fluctuations, it had grown and flourished until at the present time it had every prospect of enjoying a great and successful future. Two legacies that had been received in the past year had enabled them to pay off the mortgage on the property.

The report showed an increase in work, so that, although the total subscriptions and donations were fully maintained, there was a deficit of £52. The number of in-patients received during the year was 378, and as only about one-third of these were in a position to pay any part of their cost, it will be seen that the hospital, though described as a hospital, is rapidly becoming a free institution, entailing a marked decrease of income. A donation or £1,500 was received from King Edward's Hospital Fund which was accompanied by a letter, consequent upon the report of their visiting committee, and stated that of the donation £500 each was to be allotted to the discharge of existing debts, for general purposes, and for the building fund. The visitors pointed out that the wards were nearly always full, but far too small, the cubic capacity being only 900 feet for each adult, and recommended that this state of things should be remedied when possible. It was also thought that the committee of management should make efforts to obtain funds enabling another wing to be added to the hospital which would accommodate patients below and nurses in the upper storey. This would materially relieve the pressure on the space at the disposal of the staff, and be much more wholesome for the nurses. More space was also required for the accommodation of the nurses. The visiting committee recognised the disadvantages under which the hospital laboured, and were anxious to see it taking a more prominent position in the hospital world, and eventually becoming a free hospital. An urgent appeal is made by the Governors for funds to enable them to add to their limited accommodation in order to cope with the rapid increase of the population in the districts which it serves, a population that already numbers 700,000 within a radius of 2½ miles.

